# High rates of burnout among maternal health staff at a referral hospital in Malawi: A cross-sectional study

**DOI:** 10.1186/1472-6955-10-9

**Published:** 2011-05-23

**Authors:** Viva Combs Thorsen, Andra L Teten Tharp, Tarek Meguid

**Affiliations:** 1 Department of Community Medicine, University of Oslo, Oslo, Norway; 2 Menninger Department of Psychiatry & Behavioral Sciences, Baylor College of Medicine, Houston, USA; 3 Department of Obstetrics and Gynecology, Bwaila Hospital and Kamuzu Central Hospital, Lilongwe, Malawi

## Abstract

**Background:**

Burnout among maternal healthcare workers in sub-Saharan Africa may have a negative effect on services provided and efforts to mitigate high maternal mortality rates. In Malawi, research on burnout is limited and no empirical research has been conducted specifically among maternal health staff. Therefore, the aims of the study were to examine the prevalence and degree of burnout reported by healthcare workers who provide antenatal, intrapartum, and postnatal services in a district referral hospital in Malawi; and, to explore factors that may influence the level of burnout healthcare workers experience.

**Methods:**

In the current cross-sectional study, levels of burnout among staff working in obstetrics and gynaecology at a referral hospital in Malawi were examined, in addition to individual and job characteristics that may be associated with burnout.

**Results:**

In terms of the three dimensions of burnout, of the 101 participants, nearly three quarters (72%) reported emotional exhaustion, over one third (43%) reported depersonalization while almost three quarters (74%) experienced reduced personal accomplishment.

**Conclusions:**

Based on these findings, burnout appears to be common among participating maternal health staff and they experienced more burnout than their colleagues working in other medical settings and countries. Further research is needed to identify factors specific to Malawi that contribute to burnout in order to inform the development of prevention and treatment within the maternal health setting.

## Background

Throughout Africa the human resource crisis has severely debilitated health systems and the quality of services delivered. As a result, the existing healthcare workforce often carries excessive and sometimes complex workloads [1]. For example, in Malawi the physician-patient ratio is one physician per 62,000 compared to the World Health Organization's (WHO's) recommended ratio of one per 5000. Moreover, nursing vacancies stand at 65% [2]. Working in these environments has potential for substantial workforce burnout resulting in impaired performance, negative attitudes, illness, absenteeism, and turnover [3-5].

Burnout is a psychological term for the negative response to chronic job-related emotional stress. In other words, burnout results from people giving too much of their time, energy, and effort on the job over a long period of time without adequate time to recover physically or emotionally [6]. The prevalence of burnout among physicians ranges from 25 to 60%, and occurs at a level sufficient to affect personal or professional performance [7,8]. Among nurses/midwives 15 - 85% have reported burnout [9-12]. The prevalence varies by medical specialty and working conditions [7,13]. When comparing nurses to physicians or other healthcare workers, nurses consistently reported higher levels of burnout [14,15].

Burnout among healthcare workers detracts from quality and quantity of services and care rendered which may subsequently contribute to poor patient outcomes and health infrastructures [3,7,16-18]. Additionally, it may exacerbate the challenges of achieving Millennium Development Goal #5 (MGD 5) which is to reduce the maternal mortality ratio by 75% between 1990 and 2015 [19]. Because healthcare workers are critical to health and development, in general, and maternal health, in particular, their wellbeing is of paramount importance in the effective provision of antenatal, intrapartum and emergency obstetric care (EmOC), and postnatal services and care. Burnout represents a threat to this wellbeing.

In Malawi, there is a dearth of information on burnout, and only two studies have been conducted. One focused on how socioeconomic changes and increased workload of caring for chronically ill and terminally ill patients impacted the nurses' role [20] while the other examined the degree to which organizational attributes were associated with burnout, job satisfaction, motivation, and performance of mid-level providers in three districts in Malawi [21]. Peltzer reported that 90% of the participating nurses reported having burnout symptoms. More than half (53%) were so seriously affected that their work performance suffered and their own health was at risk. McAuliffe and colleagues found out that one third of their participants were burned out due to high levels of emotional exhaustion and more than one quarter due to low levels of personal accomplishment. These two studies cut across various healthcare departments and highlight the acute human resource crisis in Malawi. However in general, information about burnout among maternal health staff remains scant. Therefore, the current study had two aims: To examine the prevalence and degree of burnout reported by healthcare workers who provide antenatal, intrapartum, and postnatal services in a district referral hospital in Malawi; and, to explore factors that may influence the level of burnout healthcare workers experience.

## Methods

### Design

The descriptive cross-sectional study was carried out at a district referral hospital, specifically in the antenatal, labour and delivery, and postnatal units in Malawi from December 2008 to May 2009. The independent variables were individual and job-related characteristics, and the dependent variable was burnout.

### Participants

A purposive sample of healthcare workers was recruited. The inclusion criterion was healthcare workers who worked in the Department of Obstetrics and Gynaecology and related units (i.e., antenatal, postnatal units, and the theatre) at either hospital. A total of 101 healthcare workers participated in this cross-sectional study. All eligible participants who were approached agreed to participate (100% response rate). However, because some workers were absent at the time of recruitment it is estimated that 90% of the total eligible staff participated.

### Data collection and instruments

Data collection took place between the months of December 2008 and May 2009. Data were initially collected by the first author (VCT) and subsequently by a medical intern and a research nurse. The three data collectors described in detail the purpose and procedures of the study, obtained verbal consent, and gave the participants a self-administered survey. The questionnaires were confidential and all participants were assured that no individuals would be identified. The survey was comprised of the following questionnaires:

An adapted version of the Maslach Human Services Demographic Data Sheet [22], which contained questions related to demographics and job related questions such as primary work area, number of hours per week, number of hours within current job and total years within the profession.

The Maslach Burnout Inventory - Human Services Survey (MBI), which is a 22-item questionnaire that relates to three constructs of burnout: Emotional Exhaustion (EE, 9 items) captures the experience of having one's emotional resources depleted and having no source of replenishment. EE subscale items describe feelings of being emotionally overextended and exhausted by one's work (e.g. I feel like I'm at the end of my rope). Depersonalization (DP, 5 items) describes the experience of becoming cold and indifferent to other's needs. DP subscale items capture negative and cynical feelings about one's patients or colleagues (e.g. I don't really care what happens to some recipients). Reduced Personal Accomplishment (PA, 8 items) is a sense of inadequacy about one's ability to relate to patients which may result in a self-imposed verdict of "failure". PA subscale items assess how one perceives his or her competence (e.g. I deal very effectively with the problems of my recipients).

Respondents rated each item on a seven-point Likert-type scale for how frequently they experience the feeling (0 = "Never", 6 = "Every day"). For clarification, three terms were reworded or defined on the questionnaire. "Recipients" was written as "patients"; "callous" was defined as "mean/hard/insensitive", and "exhilarated" was defined as "refreshed/alive/cheerful". The scale is scored by calculating subscale means [22]. Using cut-off scores for the means based on data from a normative sample of 1104 health professionals in the United States (Table 1), respondents are classified as high, moderate or low burnout cases on the respective subscales. High mean scores on EE and DP subscales correspond to higher degrees of experienced burnout, whereas a low mean score on the PA subscale corresponds to a higher degree of burnout. The MBI yields three, noncumulative scores [22].

**Table 1:** MBI subscale cut-off scores and categories

Subscale^1^	Category	Cut-off Scores
EE (Score: 0 - 54)	High	≥ 27
	
	Moderate	19 - 26
	
	Low	0 - 18

DP (Score: 0 - 30)	High	≥ 10
	
	Moderate	6 - 9
	
	Low	0 - 5

PA (Score: 0 - 48)	High	0 - 33
	
	Moderate	34 - 39
	
	Low	≥ 40

^1 ^EE = Emotional exhaustion, DP = depersonalization, PA = reduced personal accomplishment

Maslach, Jackson and Leiter reported reliability of the MBI. Past work reported Cronbach's alpha coefficients for the subscales to be 0.90 for EE, 0.79 for DP and 0.71 for PA [22] with test-retest reliabilities ranging from 0.50 to 0.82 for the three subscales. Both convergent and discriminant validity of the MBI have been established [22,23]. The Cronbach's alpha coefficients for this study were: 0.67 for EE, 0.42 for DP, and 0.60 for PA.

Having been established over 20 years ago and cited by more than 500 studies, the MBI is considered the gold standard questionnaire in the area of burnout. It has been used throughout the United States and Europe, and more recently in Asia, Latin America, and Africa. In Nigeria, Ghana, and Malawi levels of burnout were assessed among nurses, doctors, and mid-level providers such as medical assistants, clinical officers and enrolled nurses [14,21,24,25]. In addition to assessing burnout levels among this medical cadre, psychologists', teachers', and policemen's burnout levels have been investigated in South Africa [7,26-30]. Hwang, Scherer and Ainina reviewed the factorial stability of the MBI across cultures and languages. They concluded that 'there is evidence to support the MBI as a useful measurement tool across a wide-array of languages, countries, and occupations. Because the MBI had been used previously in Malawi, not to mention in other African settings, we anticipated that its use would be appropriate for the current study. However, the low alphas we obtained may suggest modifications may be needed to maintain the scales' internal consistency in Malawi.

### Data analysis

In addition to the high category, our definition of burnout included those in the moderate group because it too poses a risk for negative outcomes and may be an indicator for prevention and intervention services [31]. Statistical Package for Social Science (SPSS) 18.0 was used to analyze the data. Analyses were descriptive and exploratory. For bivariate analyses, Pearson correlations were used for continuous variables and analyses of variance (ANOVAs) were used for categorical data. For variables with only two categories being compared, the Student's t-test for independent samples was used. Following descriptive analyses, a stepwise regression was performed to determine which factors predicted burnout. Factors that were significantly associated with burnout in the correlations or ANOVAs were included as independent variables. An α of .05 was employed for all analyses.

### Ethical Issues

This study was carried out in compliance with the Helsinki Declaration. Ethical approval was granted by The College of Medicine Research Ethics Committee in Malawi (Proposal No. 10/08/703) and The Regional Committee for Medical and Health Research Ethics in South-Eastern Norway (2008/16105). In addition, permission to conduct the study was obtained from the district health officer, director of the hospital, and senior head nurses at both sites.

## Results

### Demographics

A total of 101 healthcare workers participated in the study. The majority were Malawian (95%), female (84%) and had an average age of 38 (range = 22 - 66, SD = 11). Sixty-nine percent were married, having been married on average 13 years (range = 1 - 44, SD = 9). A large proportion was Protestant (44%), followed by those being Catholic (30%), and Seventh Day Adventist (14%). For the 69% who stated having a child, the average number of children was three (range = 1 - 7, SD = 1). On average, respondents had 15 years of education (range = 10 - 18, SD = 1.8), 12 years in the profession (range = < 1 - 43, SD = 9.5), and 8 years in their current position (range = < 1 - 32, SD = 8). Participants worked approximately 41 hours per week (range = 6 - 120, SD = 14). The overtime hours were not assessed in the current study, but anecdotal reports and past research suggests that working overtime is commonplace [32]. Based on administrative data from the study hospitals, participants usually work between 11 and 17 extra hours per week due to the acute human resource crisis (Personal Communication: Meguid, T. Fwd: clearance changes URGENT [Internet]. Message to: Viva C. Thorsen. 2010 Mar 6, 6:26 am [cited 2010 Mar 6]).

The majority of the participants were enrolled nurses (n = 64, 63%). Their job titles corresponded to enrolled nurse midwives, nurse midwife technicians, technical officers and community health nurses. Twenty-two percent (n = 22) were registered nurses, which included nursing officers, nursing sisters and registered nurse midwives. Fourteen percent (n = 14) were those within the medical cadre, which included clinical officers, medical assistants, medical interns and physicians. The majority of the participants were from the labour ward (34%), followed by the antenatal clinic (18%) and the theatre/operating room (15%).

### MBI scores

Using Maslach's three categories of burnout, 72%, 43%, and 74% of respondents experienced moderate and high levels of burnout via EE, DP, and PA subscales, respectively (Table 2). According to Maslach high burnout category, 35 participants were burned out in only one of the subscales, 31 were burned out in two subscales, three were burned out in all three categories, while 31 had no high category on any of the scales, and one participant's number of highs (either one or none) could not be determined. In sum, 69 out of the 101 participants would be classified as experiencing a high level of burnout in at least one of the subscales.

**Table 2:** Frequency percentage distribution among the three subscales according to Maslach's categorization

Emotional exhaustion			Depersonalization			Reduced Personal Accomplishment		
**Low** ≤ 18	**Moderate** 19-26	**High** ≥27	**Low** ≤ 5	**Moderate** 6-9	**High** ≥ 10	**Low** ≥40	**Moderate** 39-34	**High** ≤ 33

28 (27.7%)	40 (39.6%)	33 (32.7%)	58 (57.4%)	20 (19.8%)	23 (22.8%)	50 (49.5%)	24 (24.2%)	25 (25.3%)

95% CI 19.0%-36.5%	95% CI 30.1% - 49.1%	95% CI 23.5% - 43.8%	95% CI 47.8% - 67.1%	95% CI 12.0% - 21.6%	95% CI 14.6% - 31.0%	95% CI 40.7% - 60.4%	95% CI 15.8% - 32.7%	95% CI 16.7% - 33.8%

The mean scores, along with the ranges and standard deviations for the MBI subscales are presented in Table 3. As a group the participants reported moderate burnout on the three subscales.

**Table 3:** MBI subscale mean scores^2^

Subscale	Mean	Range	Std. Dev
EE	23.1 (Moderate)	0-46	9.7

DP	6.2 (Moderate)	0-21	4.8

PA	37.8 (Moderate)	9-48	7.5

^2 ^Sample size: EE 101, DP 101, PA 99

### Correlation among individual and job characteristics and MBI subscale scores

The correlations between continuous variables and dimensions of burnout are presented in Table 4. DP was positively associated with age, number of children, and number of years in the profession. PA was negatively associated with age, number of years married, children, years in the current job, and in the profession. No variable was significantly associated with EE.

**Table 4:** Pearson correlations of continuous demographic variables with MBI subscale scores^3^

	Emotional Exhaustion	Depersonalization	Personal Accomplishment
Age	-.09	.21*	-.27**

Religiosity	-.08	.12	.03

Years married	-.06	.17	-.30**

Number of children	.01	.23*	-.32**

Years of schooling	.09	-.02	.12

Hours per week^4^	-.06	-.09	.03

Years in current job	.06	.10	-.22*

Years in the profession	.00	.20*	-.21*

^3 ^*p < .05 **p < .01

^4 ^Hours per week refers only to hours worked in primary position and does not take into account overtime.

No ANOVA or t-test results were significant, indicating that the following variables were not significantly related to any of the MBI subscales: sex, nationality, marital status, job title, and primary work area.

### Regression analysis

Stepwise regressions were performed to explore which individual and job characteristics contributed to the explanation of the MBI subscale scores. Potential predictors were the variables that met a significant threshold in preliminary bivariate analyses. As previously stated, no variables were significantly associated with the emotional exhaustion subscale. The following variables were entered into a stepwise regression to predict depersonalization: age, number of children, and years working in the healthcare profession. The overall regression was significant, F_1,89 _= 5.05, p = .03 (Adjusted R^2 ^= .04), and number of children was the only significant predictor, β = .23, p = .03. For personal accomplishment the following variables were independent variables: age, years, married, number of children, years in current job, and years in healthcare profession. The overall regression was significant, F_1,79 _= 8.90, p = .004 (Adjusted R^2 ^= .09), and number of children was the only significant predictor, β = -.32, p = .004.

## Discussion

The first aim of this study was to determine the degree of burnout experienced by healthcare staff that provides service and care to pregnant, labouring, and postnatal women in Malawi. Across MBI subscales, numerous participants experienced burnout in the moderate and high levels in all three subscales (EE: 72%; DP: 43%; PA: 74%). When comparing those in the high category alone, the prevalence among our participants was higher than what has been reported in previous studies [33-36]. When compared to one of the Malawian studies, slightly more of our sample experienced EE (33% vs. 31%), an even larger percentage for DP (23% vs. only 5%). The largest percentage difference between the two study groups was for PA (50% of our sample vs. 27% of McAuliffe and colleagues' sample experienced PA) [21]. The finding could suggest that our participants felt less competent, and thus unsuccessful, than the mid-level providers in McAuliffee and colleagues' sample. In the other Malawian study, Peltzer reported that 90% of the nurses complained about burnout symptoms of which 53% were so seriously affected that their work performance suffered [20]. Unfortunately the MBI was not used in that study making it difficult to compare findings directly.

Mean scores (EE: 23.1; DP: 6.2; and PA: 37.8) were similar to those obtained for American medical workers, which included both nurses and physicians (22.2, 5.2, and 36.5). Similarly, Hayter reported mean scores for HIV/AIDS specialist community nurses; however a lower EE score was reported (19.2) [10]. In general, our data suggest that maternal health staff might be at a higher risk of burnout than their colleagues working in other areas. However, the classification of the group by the Maslach cut-off points must be interpreted with caution because these points tend to vary by country based on social and cultural reasons [37]. Therefore, these cut-off points have yet to be established for Malawian healthcare workers, which warrant further research.

The second aim of the paper was to determine whether or not there were any characteristics that were significantly associated with the level of burnout that participants experienced. Of the characteristics analyzed, five appeared to be associated with burnout: age, number of years married, number of children, number of years in current position and total number of years in the profession. However, in the stepwise regressions, number of children was the only significant predictor for DP and PA.

It can be inferred that domestic duties, such as child rearing and being accountable to one's spouse, may introduce additional and sometimes intensive responsibilities. The preoccupation with providing and caring for one's child and or completing domestic duties, with possibly low spousal support, may create distractions that thwart job performance. This illustrates family-to-work conflicts where the demands from the family/home and work domains are mutually incompatible such that fulfilling the parental role makes it difficult to perform the work role satisfactorily [38-41]. This may manifest in the expression of cynicism or feelings of professional inefficacy [42]. Our finding is consistent with a previous study that showed that as the number of children respondents had increased so did evidence of increasing burnout for the dimension of PA [34]. Demir and colleagues reported that problems in childcare were significantly associated with EE and PA, adversely influencing Turkish nurses participating in their study [43]. They also cited another study where having problems related to family and childcare remained stressors for public health nurses. Like these studies, our findings suggest that the hospitals should be concerned with family-work conflict as a source of burnout and as a potential liability in terms of quality of care. Future studies may explore other characteristics of family life that influence burnout, such as social support and quality of parent-child relationships.

Past work supports the assertions that gender, being a nurse, and years of education are associated with burnout [10], so it was unexpected that none of these variables were significantly associated with emotional exhaustion in our study. This may be due in part to EE being strongly correlated to excessive workload, intensity of contacts with patients, patient-to-healthcare worker ratio, and shift-working [44-48]; all of which were not collected or analyzed in this study. Another possibility is that rates of moderate and high EE were quite high in the current study (72%) which may have limited the variance in this sample, and therefore, the ability to find variables that distinguished workers with EE and those without.

Limitations inherent to the study merit discussion. First, the sample size was small and the population narrowly defined. However, given the fact there is a small number of permanent staff employed primarily in maternal health within the department of obstetrics and gynaecology in question, it was unavoidable. The focus on maternal health staff should not discount the possibility of obtaining similar findings in other departments. Nevertheless, a more diverse population, cross cutting various departments and medical settings in Malawi may increase the generalizability of the findings. In addition, a number of interviews or focus groups with participants and/or key informants might have helped add depth to the findings.

Second, the assessment of overtime hours and its association to burnout was not performed. They could explain some of the varying levels of burnout [5,49]. Intuitively, working overtime in the healthcare setting potentially increases the caseload size, time spent in direct patient contact, and encounters of unmanageable complications. In our setting, administrative data suggested 25% of the healthcare workers work an extra 17 hours per week, which translate to 75 hours month and only one night of sleep per month (Personal Communication: Meguid, T. Fwd: clearance changes URGENT [email]. Message to: Viva C. Thorsen. 2010 Mar 6, 6:26 am [cited 2010 Mar 6]). This may lead to greater emotional exhaustion depersonalization and personal accomplishment.

The current study was exploratory in nature and as a first step, the MBI was used. However, the low Cronbach's alpha values (.67 for EE, .42 for DP, and .60 for PA) suggest that the MBI may not be as culturally appropriate as assumed. Future studies will have to resolve this limitation by, for example, adding more items to the respective scales and performing item and factorial analyses. Moreover, the use of additional instruments, such as organizational commitment questionnaire, job satisfaction survey, workload scale, or interpersonal conflict at work scale to supplement the MBI will enrich future analyses and provide more alternative explanations for what is and is not found [33,50].

Last, the data were cross-sectional and collected from self reports which do not allow for causal conclusions. Individuals with high negative affectivity may perceive their work context more negatively, which would artificially strengthen the associations between burnout symptoms and work environment.

## Conclusions

Though circumscribed in scope and limited by a number of methodological issues, this study contributes to the research on burnout and documents that there was significant burnout morbidity among participating maternal health staff. This study is a critical step to begin addressing some gaps in the research. Because the concept of burnout is still fairly new in Malawi, its seriousness may not yet be fully understood or appreciated. However, burnout is an important barometer of major dysfunctions in the workplace. It may be symptomatic of deeper systemic problems within a given setting [33,51]. Therefore, burnout may have serious implications not only for the quality of emotional, technical, and emergency obstetric care given to women but also for the health and wellbeing of the maternal healthcare workers themselves.

## Competing interests

The authors declare that they have no competing interests.

## Authors' contributions

VCT was responsible for the study conception and design and undertook part of the data collection. VCT and ATT were responsible for data analysis. VCT was responsible for drafting the manuscript. VCT, ATT, and MT made critical revisions of the paper. All authors read and approved the final manuscript.

## Pre-publication history

The pre-publication history for this paper can be accessed here:

http://www.biomedcentral.com/1472-6955/10/9/prepub
